# Another Hint to the Oxford Infirmary

**Published:** 1893-02-11

**Authors:** 


### CONSTRUCTION NOTES.

### ANOTHER HINT TO THE OXFORD INFIRMARY.

The difficulties in the way of reflooring the worst of the wards of Addenbrooke's Hospital, Cambridge, to which we alluded in our last number, have been overcome by the opportune assistance of a member of the medical staff. At a special Court of Governors held on February 6th, it was unanimously resolved that in consideration of special donations to the amount of £300, collected from Cambridge ladies by Dr. Donald MacAlister within the last three weeks, the floors of Bowtell, Griffith, and Albert wards should be entirely relaid with teak in the best manner under the supervision of Mr. W. M. Fawcett, architect. A tender to complete the work by March 31st for £571, sent in by Redding and Sons, was thereupon accepted, and the thanks of the Court were enthusiastically voted to Dr. MacAlister and to those who had so generously come forward to help in putting the hospital above reproach from a sanitary point of view.

